# Cell Adhesion Molecules and Their Roles and Regulation in the Immune and Tumor Microenvironment

**DOI:** 10.3389/fimmu.2019.01078

**Published:** 2019-05-22

**Authors:** Heidi Harjunpää, Marc Llort Asens, Carla Guenther, Susanna C. Fagerholm

**Affiliations:** Research Program of Molecular and Integrative Biosciences, Faculty of Bio- and Environmental Sciences, University of Helsinki, Helsinki, Finland

**Keywords:** cell adhesion, integrin, LFA-1, ICAM-1, VCAM-1, immunotherapy, dendritic cell (DC)

## Abstract

The immune system and cancer have a complex relationship with the immune system playing a dual role in tumor development. The effector cells of the immune system can recognize and kill malignant cells while immune system-mediated inflammation can also promote tumor growth and regulatory cells suppress the anti-tumor responses. In the center of all anti-tumor responses is the ability of the immune cells to migrate to the tumor site and to interact with each other and with the malignant cells. Cell adhesion molecules including receptors of the immunoglobulin superfamily and integrins are of crucial importance in mediating these processes. Particularly integrins play a vital role in regulating all aspects of immune cell function including immune cell trafficking into tissues, effector cell activation and proliferation and the formation of the immunological synapse between immune cells or between immune cell and the target cell both during homeostasis and during inflammation and cancer. In this review we discuss the molecular mechanisms regulating integrin function and the role of integrins and other cell adhesion molecules in immune responses and in the tumor microenvironment. We also describe how malignant cells can utilize cell adhesion molecules to promote tumor growth and metastases and how these molecules could be targeted in cancer immunotherapy.

## Introduction

### Cancer and the Immune System

The immune system and cancer cells share complex interactions during tumor development. Indeed, all types of immune cells can be found in different tumors (1–3). These include lymphocytes such as T and B cells and natural killer (NK) cells, myeloid cells such as dendritic cells (DCs) and macrophages and granulocytes such as neutrophils, eosinophils, and mast cells. The immune contexture, or the frequency, location and functional orientation of different immune cell subsets, varies substantially between tumor types and also between individuals with seemingly identical cancers (2). Interestingly, correlations between immune contexture in the tumor microenvironment and clinical outcomes have been examined in various malignancies. In general, a strong infiltration of memory CD8^+^ T cells and T helper 1 (Th1) cells correlates with favorable prognosis while strong T helper 2 (Th2) or T helper 17 (Th17) orientation is associated with poor prognosis in terms of overall survival (2, 3). In addition, high infiltration of regulatory cells such as regulatory T cells (Tregs) and myeloid-derived suppressor cells (MDSCs) in tumors often correlates with decreased survival (4–6). Understanding the role of the immune system for tumor development has been the central focus of tumor immunology since its inception. It has become evident that immune cells are able to recognize and kill malignant cells and thus suppress tumor growth in a process known as cancer immunosurveillance (7). In addition to directly targeting the cancer cells, the immune system prevents viral infections and thus the growth of virus-induced tumors and inhibits tumor-promoting inflammation by eradicating pathogens and by clearing existing inflammation. However, it is now known that the immune system can also promote tumor growth by maintaining chronic inflammation, by shaping tumor immunogenicity and by suppressing anti-tumor immunity, and thus a concept called cancer immunoediting is currently preferred to cancer immunosurveillance (7).

Cancer immunoediting is a process in which both innate and adaptive immune systems work together to suppress/control and promote/shape tumor growth (8). In order for this process to take place, the DCs need to take up tumor antigens, migrate to lymph nodes, present tumor antigens to T cells in the lymph nodes and then activated T cells need to expand and traffic into the tumor site where they interact with the malignant cells. These events can eventually lead to tumor cell killing or immune cell exhaustion. Cell adhesion molecules such as integrins and receptors of the immunoglobulin superfamily play a crucial role in all these events.

### Cell Adhesion Molecules (CAMs)

Adhesion molecules are generally divided into five groups: integrins (discussed in greater detail below), selectins, cadherins, members of the immunoglobulin superfamily (IgSF) including nectins and others such as mucins (9). In addition to the conventional adhesion molecules, certain enzymes such as vascular adhesion protein 1 (VAP-1) also play a role in cell adhesion (10).

Apart from structural differences, cell adhesion molecules also bind to different ligands. Integrins typically bind to the extracellular matrix, while selectins, cadherins, and IgSF members are associated with cell-cell adhesion (9). However, immune cell integrins also bind to soluble ligands and ligands on other cells. The cell-cell adhesion mediating molecules can further be divided by their ligands as selectins bind carbohydrates in a calcium dependent manner (11), cadherins mediate preferably homophilic bonds in a calcium-dependent manner (12) and the IgSF subfamily nectins mediate homophilic and heterophilic bonds (9).

Selectins are further divided into P-, E- and L-selectins originally based on which cell types they were found in: **p**latelets, **e**ndothelial cells and **l**eukocytes (however, P-selectin is also expressed on endothelial cells) (13). Selectins differ in kinetics of expression, as P-selectins are expressed within minutes and E-selectins within hours (13). Selectins are especially important for leukocyte trafficking, migration of lymphocytes to peripheral lymph nodes and to the skin. Their most prominent function is associated with the initial stage of the rolling cell adhesion cascade in which selectin binding enables rolling (14). Selectin binding also initiates the subsequent integrin dependent step of slow rolling and cell arrest as selectin binding together with chemokine receptor activation initiates inside out signaling leading to integrin activation (see later sections) (13, 15). L-selectins in particular display a force dependent binding e.g., L-selectin forms catch bonds with its ligands (bonds that strengthen under force). Catch bonds dominate until the applied force reaches a force threshold upon which slip bonds are formed (bonds that weaken under force) (13, 14). In other selectins this occurs to a lesser extent (13, 14).

Proper functionality of selectins is carbohydrate-dependent as is demonstrated in a disorder called leukocyte adhesion deficiency II (LAD II). LAD II is caused by a mutation in a fucose transporter protein leading to fucose not being incorporated into selectin ligands, which ultimately leads to disruption of selectin-mediated leukocyte adhesion (13). Many selectin ligands, including P-selectin glycoprotein ligand 1 (PSGL-1) which is the main ligand for all three selectins (13), mucosal addressin cell adhesion molecule-1 (MAdCAM-1) and peripheral node addressin (PNAd) belong to a glycoprotein family called mucins which function as major components of the mucous protecting epithelial cells lining the digestive, respiratory and urogenital tracts (16). Interestingly, over-expression of mucins, MUC-1 in particular, have been detected in many human malignancies and seem to promote cancer cell growth and survival (17).

Cadherins are associated with cell-cell adhesive bonds in solid tissues (12). Molecules of this family feature cadherin repeat sequences which are stabilized by calcium ions. The essential role of calcium for cadherin adhesive function is also reflected in the protein family name which is an abbreviation of “**ca**lcium-**d**ependent ad**her**ent prote**ins**” (12). Cadherins in turn can be subdivided into classical cadherins (type I and II), protocadherins and atypical cadherins (9, 12).

The IgSF is one of the largest and most diverse protein families (18). All members of the IgSF contain at least one immunoglobulin or immunoglobulin-like domain and most members are type I transmembrane proteins with an extracellular domain (containing the Ig domain[s]), transmembrane domain and a cytoplasmic tail. The most well-known members include major histocompatibility complex (MHC) class I and II molecules and proteins of the T cell receptor (TCR) complex (18). Intercellular adhesion molecules (ICAMs), vascular cell adhesion molecules (VCAMs), MAdCAM-1 and activated leukocyte cell adhesion molecule (ALCAM), which are important in leukocyte trafficking events, also belong to this family of adhesion receptors (19–21). Of interest, MAdCAM-1 contains both Ig and mucin domains placing it as a member of both IgSF and mucin families (19).

Yet another subfamily of the IgSF is the nectin family which members mediate cell-cell adhesion in various tissues including endothelium, epithelium and neural tissue (9). The members can form homophilic interactions with each other or heterophilic interactions with other nectins or other ligands. They can also co-operate with cadherins to establish adherens junctions (9, 22). From an immunological point of view, interactions between nectins and immune modulatory receptors such as DNAM-1 (CD226) and TIGIT are of particular interest due to their involvement in regulation of effector cell function and their recently appreciated role in anti-tumor responses (23). Interestingly, the expression of nectins is also often up-regulated in various tumors (9).

### Integrins

Integrins are large heterodimers consisting of α- and β-chains that together form the intact receptor in the plasma membrane. Integrins bind to a wide variety of ligands in the extracellular matrix, on the surface of other cells and also soluble proteins. Leukocytes express various integrins while the β1-integrins (α4β1), β2-integrins (αLβ2, αMβ2, αXβ2, αDβ2), and β7-integrins [α4β7 and αE(CD103)β7] playing particularly important roles in immune cells.

β2-integrins are the predominant integrins on leukocytes (24). The different integrins in this family have different expression in different leukocyte subpopulations (25, 26). LFA-1 (αLβ2, CD11a/CD18) is expressed in all leukocytes and is the predominant integrin in lymphocytes. Mac-1 (αMβ2, CD11b/CD18, CR3) dominates on myeloid leukocytes, especially neutrophils, but is also expressed in NK cells, B cells and some T cells, whilst αXβ2 (CD11c/CD18, CR4) is most abundant on myeloid dendritic cells (DCs). αDβ2 (CD11d/CD18) is the most recently discovered β2-integrin and is expressed on neutrophils, monocytes and NK cells. LFA-1-integrin is more distantly related than the other β2-integrin family members and has a more restricted ligand binding profile compared to the other β2-integrins. It mainly binds members of the ICAM-1-5 and JAM-1 families. In contrast, Mac-1-integrin has a very broad ligand repertoire, with more than 40 reported ligands including ICAM-1-4, iC3b, fibrinogen, fibronectin, factor X, heparin, polysaccharides, and even denatured proteins (26). αXβ2 and αDβ2 are more closely related to Mac-1 than to LFA-1 and have similar, but more restricted, ligand binding properties than Mac-1.

The extracellular domains of integrins are large and consist of several domains (27). In those integrins that contain it, the αI (or A) domain is the most important ligand binding domain, and mediates Mg^2+^-dependent ligand binding. The I-domain consists of a central β-sheet surrounded by seven α-helices. Ligand binding happens at the MIDAS-site which provides three surface loops to co-ordinate the Mg^2+^ ion, whilst a glutamate or aspartate from the ligand provides the fourth coordination position. This is referred to as the I-domain open conformation. In the closed conformation, the fourth coordination position is replaced by a water molecule – this induces structural changes in the I-domain e.g., rearrangement of the metal-co-ordinating residues and a 10Å shift in the α7-helix. Ligand binding shifts the equilibrium from the closed towards the open state (27).

The I-domain forms part of the ligand binding head domain of the integrin extracellular domain, which also contains the βI-domain (that has a similar structure as the αI domain) and the propeller domain in the α-chain (27). The I-domain sits on top of the propeller domain. Structural signals can be transduced through the integrin β-chain to induce conformational changes in the integrin head-domain or from the ligand-bound head into the cell (27).

**Figure 1:**
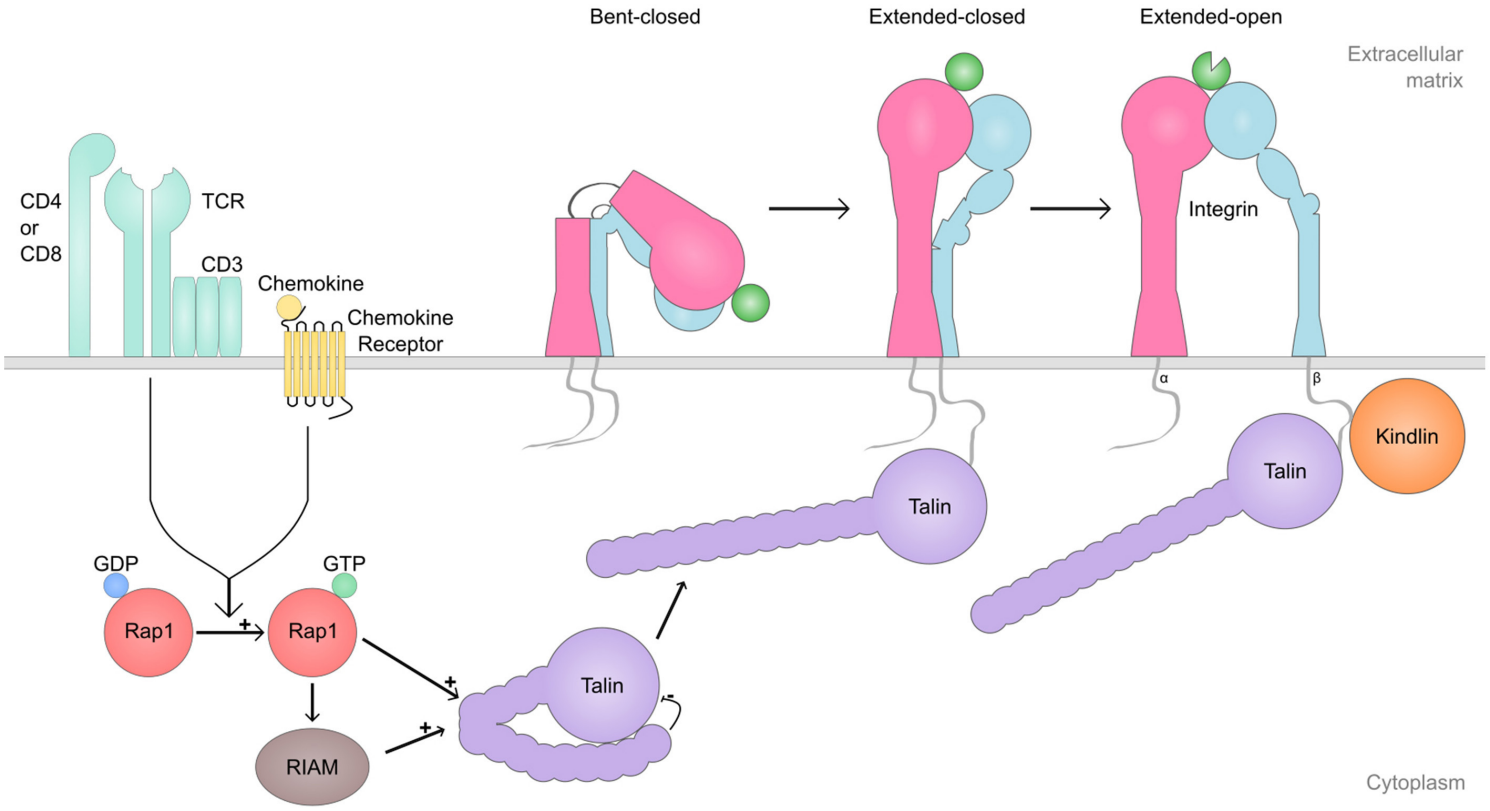
Integrin inside-out signaling. Shown is a simplified representation of the integrin inside-out process, which regulates integrin activation (i.e., integrin conformation switch from bent-closed or extended-closed to extended-open conformation). Cell signaling initiated by receptors such as chemokine receptors, T cell receptor (TCR), Toll-like receptors (TLR), and selectins, among others, trigger the switch from Rap1-GDP to Rap1-GTP which, either dependently of RIAM or not, activate Talin and enable its binding to the β-cytoplasmic tail of the integrin. Finally, Kindlin binds to the β-cytoplasmic tail of the integrin, and together with talin induces the separation of the cytoplasmic tails, and triggers the activation of the ligand-binding domain. The extended-open conformation of the integrin remains stable with Talin and Kindlin bound.

In addition to I-domain conformational changes in the ligand-bound and unbound states, integrins can undergo much larger scale structural changes (27–29) (Figure 1). When the first integrin ectodomain was crystallized, it was a surprise that the integrin was found in a bent state, with both the α-chain and the β-chain being “bent at the knee” (genu), causing the ligand-binding head to turn down towards the legs of the integrin heterodimer. Indeed, integrins can be found in bent conformation, extended/closed conformation (where the integrin is extended but the I-domain is closed) as well as extended/open conformation (extended integrin/I-domain is in the open conformation) (28).

The transmembrane domains of integrins associate with each other at two motifs, maintaining the integrin in an inactive state (30). The intracellular domains are short and devoid of enzymatic activity. However, they are nevertheless important for regulating integrin function (see below). The cytoplasmic domains of the integrin β-chain are structurally related to each other with several important motifs that are essential for integrin regulation. In contrast, the cytoplasmic domains of the integrin α-chain are more diverse, presumably allowing different integrins to have different functional characteristics.

Inside the cell, these receptors link to the actin cytoskeleton through their cytoplasmic tails (31). In cells such as a fibroblast, they form large multiprotein complexes with intracellular molecules called focal adhesions, the composition of which have been determined by proteomic methods in recent years (31, 32). Integrins also participate in so called outside-in signaling, e.g., transmitting signals into cells through a variety of signaling pathways to change cell behavior (actin reorganization, cell migration, gene expression etc.) (33).

### Integrin Regulation

Integrins are not constitutively active and able to bind ligands. Instead, their activity is regulated from inside the cell, through a process called inside-out signaling (24) (Figure 1). During this process, cell signaling initiated by other cell surface receptors, induce integrin activation. Receptors such as chemokine receptors, TCR, Toll-like receptors (TLRs), selectins as well as many other cell surface receptors, have been reported to induce integrin activation in immune cells. Integrins can also influence the activity status of other integrins, a process called integrin transregulation (34).

Ultimately, integrin activation in response to inside-out signaling is achieved by cytoplasmic factors that interact with the integrin β-chain cytoplasmic portion (Figure 1). Talin is the most well-known integrin activator. Talin binds to the membrane-proximal NPXY motif in the β-chain and is of fundamental importance for integrin activation. Talin binding to the integrin β-chain cytoplasmic domain destabilizes the transmembrane linkage between the α- and β-chain of the integrin, allowing integrin activation (30).

Kindlin is a more recently discovered integrin interaction partner which is nevertheless very important for integrin regulation (35–37). Kindlin binds to the membrane-distal NPXY-motif and a threonine-motif between the NPXY-motifs, but exactly how kindlin regulates integrin function remains incompletely understood. It has been suggested that talin is required for the conformational change of the integrin to the extended, intermediate affinity conformation, but that both talin and kindlin-3 are required for the induction of the high-affinity conformation (38, 39) (Figure 1). However, kindlin has also been reported to play a role in integrin clustering, stabilizing the integrin-ligand contact and strengthening cell adhesion (40), by recruiting downstream components such as actin, ILK, and paxillin (41–44).

The small GTPase Rap1 also plays a critical role in integrin activation (45, 46). The effects of Rap1 on integrin activation are at least in part dependent on the Rap1 interacting protein, RIAM (47). RIAM is a Rap1 effector molecule that is important for at least β2-integrin lymphocyte trafficking from blood into peripheral lymph nodes (pLNs) (45), but not for the regulation of all integrins (β3-integrin activation in platelets is independent of RIAM). However, for β2-integrins, the pathway seems to consist of Rap1/RIAM/Talin (45, 48). Very late antigen-4 (VLA-4) (α4β1, CD49d/CD29) -mediated adhesion is dependent on talin but only partly dependent on RIAM (45, 48) suggesting that also RIAM-independent Rap1/talin pathways exist. Recent studies seem to indicate that a RIAM-independent Rap1/talin pathway is of particular importance in cells that rely on quick integrin activation to function efficiently, such as neutrophils and platelets (49).

**Figure 2:**
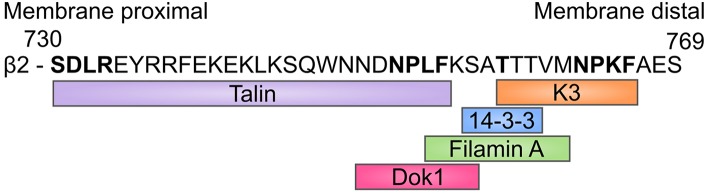
β2-integrin binding sites. Amino acid sequence of the β2-cytoplasmic tail where most of the main integrin binding proteins bind, and the sequences to which they bind. The amino acids highlighted in bold are of particular importance. 14-3-3 proteins only bind to Th758-phosphorylated integrin, whilst phosphorylation of this site inhibits Filamin A binding.

In addition to talin and kindlin, integrins interact with a multitude of cytoplasmic proteins, for example filamin A, Dok1 and 14-3-3 proteins (50) (Figure 2). Filamin A was previously thought to be a negative regulator of integrins, either by inhibiting talin binding (51) and/or by crosslinking integrin cytoplasmic domains (52). However, recent results indicate that it may instead be important for integrin-mediated shear flow adhesion and trafficking of immune cells *in vivo* (53). Integrin cytoplasmic domain phosphorylation has been reported for many integrins and plays a role in regulating interactions with cytoplasmic molecules and therefore further regulates integrin function (24).

### The Function of Integrins and Other Cell Adhesion Molecules in Immune Responses

Patients suffering from leukocyte adhesion deficiency type I (LAD-I) have lost or reduced expression of β2-integrins on their leukocytes, and these patients suffer from recurrent bacterial infections (54). Symptoms also include leukocytosis, periodontitis and delayed wound healing. In leukocyte adhesion deficiency type III (LAD-III), integrins are expressed but dysfunctional because kindlin-3 is mutated or absent, and these patients have similar symptoms as LAD-I patients (54). However, they also suffer from a Glanzmann-type bleeding disorder as kindlin-3 is required not only for β2-integrin-mediated leukocyte adhesion but also for β3-integrin-mediated platelet adhesion. These findings show that β2-integrins and their cytoplasmic regulators play fundamentally important roles in immunity (55). Studies with mice deficient for different β2-integrins have further revealed individual contributions to various leukocyte processes (56, 57).

Leukocytes traffic out of the blood stream into the lymph nodes, tissues or tumors by using the leukocyte adhesion cascade, which is regulated by sequential function of adhesion molecules (selectins, integrins, receptors of the IgSF) (58, 59). In brief, selectin-selectin ligand interactions lead to rolling of the leukocyte on endothelial cells, allowing activation of the cell by chemokines present on the endothelium. This leads to activation of integrins on the surface of the immune cell (15). LFA-1 and VLA-4 integrin activation by talin and kindlin allows firm interaction between the immune cell such as a T cell or a neutrophil and endothelial cells, which express integrin ligands such as ICAMs, VCAM-1, and MAdCAM (37, 38, 58, 60, 61). This is followed by cell spreading, Mac-1-mediated crawling (62), paracellular or transcellular extravasation, and migration into lymph nodes or tissues. In effector T cells, LFA-1 is up-regulated and constitutively activated, which contributes to the trafficking properties of these cells to peripheral tissues (63, 64). In tumors, several steps of the leukocyte trafficking process can be severely disrupted (discussed below).

Adhesion is important also in other immune cell interactions. LFA-1-ICAM-1 interaction, in particular, plays an essential role in the formation of the immunological synapse (IS) between a DC and a T cell (65–67). The structure of an IS is highly organized with key interacting molecules organized in distinct areas called supra-molecular activation complexes (SMACs) (68). The central region of the SMAC (cSMAC) is enriched in TCRs and associated molecules while LFA-1 and ICAM-1 are localized in the peripheral region of the SMAC (pSMAC) and large molecules such as CD45 and CD43 in the distal area of the SMAC (dSMAC). Also VLA-4 is localized at the pSMAC (69). Due to the crucial role for the stabilization of the immunological synapse, LFA-1 is important for T cell activation and proliferation (70, 71). In addition, talin and kindlin-3-mediated activation of LFA-1 has been shown to be important in T cell activation *in vivo* (71, 72). LFA-1 also provides a necessary co-stimulatory signal for T cells lowering the threshold for activation and proliferation following TCR engagement and promotes their IL-2 production (71, 73–75) In addition, LFA-1 has been reported to play a role in Th1/Th2 polarization, development and/or maintenance of Tregs and follicular T cells, and for generation of memory T cells (61, 76–79). Further, LFA-1-aided IS formation is important in the contact between cytotoxic CD8^+^ T cell/NK cell and the target cell such as a tumor cell and for the release of cytotoxic granules and target cell killing by CD8^+^ T cells and NK cells (61, 80, 81). Together, these studies show that LFA-1-ICAM-1 pathway plays a crucial role in T cell trafficking, activation and function and thus in the protection of the host from infections and cancer.

Mac-1 and αXβ2 are important receptors for complement iC3b thus mediating phagocytosis of complement-coated particles (82). Also neutrophil degranulation is dependent on Mac-1 integrin (56, 83). Therefore, Mac-1 is vital for the functionality of myeloid cells. However, interestingly, Mac-1 in myeloid cells can also function as a suppressor of immune responses (26) by e.g., inhibiting TLR-signaling in macrophages (84, 85). β2-integrins and the β2-integrin ligand ICAM-1 can also repress DC-mediated T cell activation (86–89) and Th17 differentiation (90, 91) and restrict DC trafficking from peripheral sites to lymph nodes (89, 92).

ICAM-1 is the main ligand for β2-integrins (24). It is expressed at basal levels on several cell types including fibroblasts, keratinocytes, endothelial cells, and leukocytes and its expression increases during inflammation due to inflammatory cytokines such as IFNγ, IL-1β, and TNFα (93, 94). In inflamed tissues, ICAM-1 expressed on endothelial cells binds to β2-integrins LFA-1 and Mac-1 on leukocytes and facilitates their transendothelial migration to the inflammation site. Given the role in mediating leukocyte migration, ICAM-1 up-regulation has been associated to various inflammatory, autoimmune and allergic diseases (94–96). Interestingly, ICAM-1 is also expressed in many types of tumors where it plays a dual role in tumor growth (discussed below).

VCAM-1 is predominantly expressed on endothelial cells and, similar to ICAM-1, its expression increases during inflammation due to inflammatory cytokines such as TNFα (97, 98). VCAM-1 is an important mediator of immune cell mediated rolling, adhesion and extravasation into inflamed tissues by binding to VLA-4 on leukocytes. Thus, VCAM-1 expression has been associated with several autoimmune disorders including rheumatoid arthritis and asthma. In addition, again similar to ICAM-1, VCAM-1 has been shown to play a dual role in cancer development (discussed below).

Integrins and integrin ligands therefore play crucial roles in several immune system functions relevant for tumor rejection, especially in immune cell migration and activation. Indeed, cell adhesion molecules have been shown to play both positive and negative roles in anti-tumor immunity.

### Cold Tumors Often Display Dysregulated Expression of Cell Adhesion Molecules on the Tumor-Associated Vessels

Based on the immune landscape, tumors can be divided into inflamed or “hot” and non-inflamed or “cold” tumors. Hot tumors are characterized by transcripts encoding for various T cell-lineage markers, innate immune cell molecules, chemokines that regulate effector T cell recruitment and also for molecules mediating immune suppression such as PD-L1, Foxp3 and indoleamine-2,3-dioxygenase (IDO) (59, 99, 100). These tumors are highly infiltrated by tumor-infiltrating lymphocytes (TILs) but their function is inhibited due to immune suppressive tumor microenvironment. By contrast, cold tumors have low expression of the before-mentioned transcripts but instead express high levels of factors associated with angiogenesis, molecules involved in Notch and/or β-catenin pathway and serine protease inhibitors. Cold tumors are generally poorly infiltrated by TILs and effective T cell homing into the tumor remains a major obstacle for endogenous anti-tumor immunity and for the success of cancer immunotherapies. Migration of immune cells into tumors can be hampered by many factors, such as impaired chemokine expression at tumor sites, mismatch between chemokines expressed at the tumor site and the set of chemokine receptors being expressed on immune cells, fibrosis around the tumors, and abnormal tumor vasculature (101). Aberrant adhesion molecule expression on leukocytes and cancer cells enables immunoediting and evasion of immune system surveillance while aberrant expression of adhesion molecules on tumor-associated blood vessels can render the whole tumor mass inaccessible for the immune system.

Indeed, down-regulation of adhesion molecules on tumor-associated endothelial cells, a process termed as endothelial anergy, is an effective mechanism utilized by tumors to prevent immune cell trafficking into the tumor site (102, 103). Down-regulation of several adhesion molecules such as ICAM-1/2, VCAM-1, E-selectin, P-selectin, and MAdCAM-1 has been reported in tumor-associated vessels in various human malignancies (104–109) which is at least partly due to high levels of angiogenic factors such as basic fibroblast growth factor (bFGF) and vascular endothelial growth factor (VEGF) in the tumor microenvironment (105, 110). It has been demonstrated both *in vitro* (111) and *in vivo* (112) that low adhesion molecule expression on the endothelial cells due to angiogenic factors leads to diminished leukocyte-vessel wall interactions. Further, diminished leukocyte interactions with tumor endothelium is caused by down-regulation of adhesion molecules on the endothelium and not by decreased expression of LFA-1, VLA-4, or L-selectin on leukocytes (110). From a clinical perspective, up-regulation of ICAM-1 in the tumor microenvironment have been shown to be related to favorable prognosis among patients with various cancers, suggesting an enhancement in cancer immunosurveillance (113–115). Indeed, an increase in TILs was observed in ICAM-1 positive gastric and colorectal cancers compared to ICAM-1 negative tumors (114–116). In addition, Tachimoro et al. showed that more human peripheral blood mononuclear cells (PBMCs) adhered to colon carcinoma (LM-H3) cells *in vitro* that were transfected with ICAM-1 compared to cells with an empty vector (117). PBMC-mediated cytotoxicity was also enhanced towards ICAM-1 expressing LM-H3 cells. Further, when injected into nude mice, a significant reduction was observed in subcutaneous tumor growth and ability to metastases liver with ICAM-1^+^ LM-H3 cells compared to ICAM-1 negative cells. In summary, expression levels of integrin ligands on tumor endothelium clearly influences anti-tumor immune responses by affecting immune cell infiltration into tumors.

### Expression of Integrins and Other Cell Adhesion Molecules Affect T Cell Infiltration Into Tumors

T cells are important for the recognition of tumor-specific antigens and for the killing of malignant cells (118, 119). Thus T cells, particularly CD8^+^ T cells, have been demonstrated to be crucial in protecting the host from malignant tumor growth (120, 121). Indeed, CD8^+^ T cell infiltration and high CD8^+^ T cell/Treg ratio in the tumor microenvironment has been associated with favorable prognosis in different human malignancies (4, 122, 123). In order for CD8^+^ T cells to mediate tumor cell killing, they must first become into contact with the malignant cells. However, T cells often fail to infiltrate the tumor tissue, thereby causing a major obstacle for successful treatment of cancer patients with immunotherapy (99, 124). The mechanism of T cell homing to healthy and infected tissues is well-understood but this process may be significantly altered during cancer.

Murine studies have demonstrated that expression levels of both integrin ligands on endothelial cells and integrins on T cells influence T cell tumor infiltration. Fisher et al. showed that ICAM-1 deficiency or blockade decreased CD8^+^ T cell infiltration into melanoma (B16-OVA) or colon carcinoma tumors (CT26), respectively, demonstrating ICAM-1 expression affecting T cell infiltration into tumors at least in certain tumor models (125). Sartor et al. observed a suppression in tumor growth rate in mice inoculated with fibrosarcoma tumor cells expressing ICAM-1 compared to mice with ICAM-1 negative tumors suggesting an increase in T cell-mediated immunosurveillance in the presence of ICAM-1 (126). Interestingly, also the expression of αE (CD103) on tumor-infiltrating CD8^+^ T cells has been shown to increase CD8^+^ T cell trafficking into tumors and thus promoting anti-tumor immunity (127). αE^+^ CD8^+^ T cells had superior capacity to accumulate in tumors in humanized mouse models of breast cancer due to the specific binding of αE on CD8^+^ T cells to E-cadherin expressed on epithelial cancer cells.

In human patients, high expression of ICAM-1, VCAM-1, and MAdCAM-1 has been shown to correlate with higher density of CD8^+^ T cells in colorectal cancer (CRC) tumors and also with prolonged disease-free survival (128). Further, human hepatocellular carcinomas (HCC) are more heavily infiltrated with T cells compared to colorectal hepatic metastases (CHM) and that the T cell infiltration was associated with higher expression of ICAM-1 and VAP-1 on tumor-associated endothelial cells in HCC (106). Additionally, a higher proportion of tumor-infiltrating T cells in both tumor types expressed LFA-1 and VLA-4 compared to peripheral blood T cells. In addition, mainly anti-ICAM-1 and anti-VAP-1 and to a lesser extent anti-VCAM-1 mAbs inhibited HCC-derived T cell binding to tumor vascular endothelium *in vitro* suggesting that LFA-1/ICAM-1 and VAP-1 receptor/VAP-1 are crucial pathways mediating T cell recruitment into the tumor site in HCC. Correlation between levels of cell adhesion molecules (ICAM-1, E-selectin, P-selectin) and T cell infiltration levels has also been shown in melanoma, glioblastoma, Merkel cell carcinoma and squamous cell carcinoma (SCC) (108, 109, 129, 130). Interestingly, when SCC samples were treated with a TLR-7 agonist, imiquimod, tumor vessels up-regulated E-selectin expression causing an increase in CLA^+^ CD8^+^ T cell influx into the tumor, a decrease in Treg frequency and tumor regression. Together, both human and murine studies indicate that integrins and VAP-1 on T cells and integrin ligands on endothelial cells are of crucial importance for T cell infiltration into tumors.

Pre-clinical mouse studies indicate that integrins and integrin ligands may also have other effects on anti-tumor responses besides T cell recruitment into tumors, by influencing T cell priming and effector functions. Schmits et al. showed that LFA-1 deficient mice had defects in CD8^+^ T cell priming against tumor-specific antigens and thus, failed to reject immunogenic fibrosarcoma tumors (57). Mukai et al. further demonstrated that administration of anti-LFA-1 mAbs abrogated the efficacy of adoptive T cell therapy in mouse models of pulmonary and intracranial fibrosarcomas (131). By contrast, ICAM-1 deficiency or mAbs targeting ICAM-1, VCAM-1, or VLA-4, showed no inhibition on the efficacy of transferred T cells in the same mouse models. Interestingly, LFA-1 blockade and to a lesser extent ICAM-1 blockade caused a decrease in T cell IFNγ production in mixed tumor/T cell cultures but did not affect the level of T cell infiltration into the tumor. These results suggested that the LFA-1 pathway mainly affects T cell effector function but not migration to the tumor site. In addition, given that LFA-1 blockade but not ICAM-1 deficiency/blockade affected the anti-tumor efficacy of adoptive T cell therapy, LFA-1 interactions with other ligands such as ICAM-2 seem to be sufficient to preserve T cell effector function. Accordingly, adoptive T cell studies conducted by Blank et al. further demonstrated that CD8^+^ T cell infiltration into tumor site is not inhibited in ICAM-1 deficient mice (132). Rather, host ICAM-1 expression affected the priming of adoptively transferred tumor-antigen-specific CD8^+^ T cells leading to delayed tumor rejection in ICAM-1 deficient mice. In addition to priming, LFA-1 and integrin αE(CD103)β7 expressed on CD8^+^ T cells play important roles for CD8^+^ T cell cytotoxicity towards tumor cells expressing ICAM-1 and E-cadherin (133).

In addition to T cell migration into tumors, T cells must also be able to recirculate from the tumor site to draining lymph nodes in order to mount distant responses (134). Interestingly, Yanguas et al. showed that in a mouse model of melanoma, increased number of intra-tumorally injected tumor-specific T cells migrated into the draining lymph nodes in mice treated with anti-ICAM-1 or anti-LFA-1 mAbs compared to mice treated with control IgG (134). Further, activated T cells formed intra-tumoral clusters mediated by LFA-1/ICAM-1 interactions in mouse models of melanoma and breast cancer and similar T cell clusters were also visible in primary human melanoma. These results suggested that LFA-1/ICAM-1 pathway also mediates a mechanism to trap activated CD8^+^ T cells in the tumor tissue.

In summary, integrins, integrin ligands and other cell adhesion molecules expressed on T cells and endothelial cells mediate CD8^+^ T cell trafficking into tumors at least in some tumor models, but may also play crucial roles in T cell priming and effector functions, thereby affecting anti-tumor immunity in a multitude of ways.

### Integrins and Other Cell Adhesion Molecules Affect DC Function During Anti-Tumor Immunity

**Figure 3:**
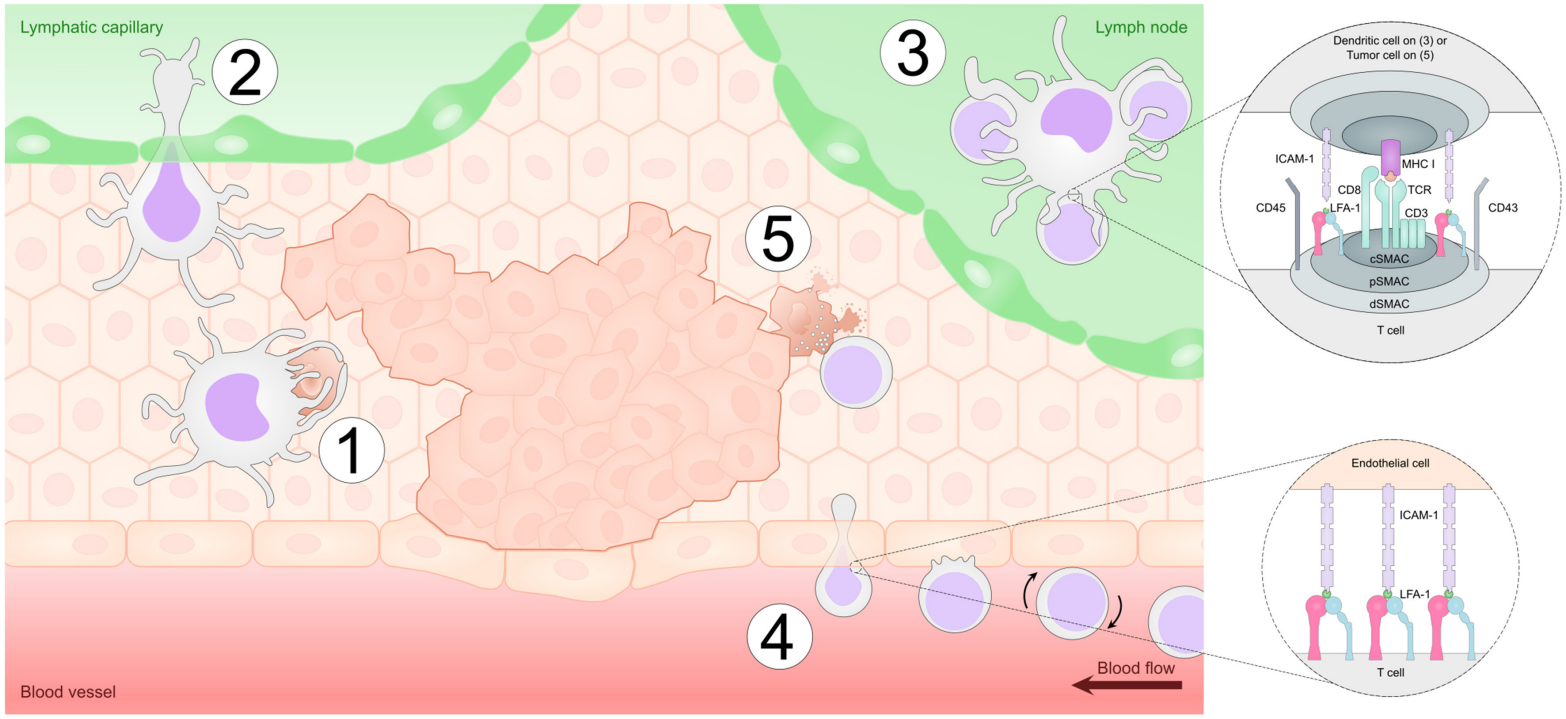
Integrins play a vital role in anti-tumor immunity. Dendritic cells (DCs) take up tumor antigens in the tumor microenvironment by phagocytosing dying tumor cells in a process mediated by adhesion molecules such as α_v_β_5_ integrins (Step 1). DCs then enter the lymphatic vessels partly in an LFA-1/ICAM-1-dependent manner and migrate to the draining lymph node (Step 2). In the lymph node, DCs form an immunological synapse with CD8^+^ T cells in order to present the tumor antigen. LFA-1-ICAM interactions mediate adhesion in the immunological synapse and also provide an additional co-stimulatory signal to the T cells (Step 3). Once activated, T cells travel via the blood stream and enter the tumor site by interacting with adhesion molecules including E-selectin, ICAMs and VCAM-1 on endothelial cells in a process termed leukocyte adhesion cascade. This process is regulated by sequential expression of selectins (L-selectin) and integrins (LFA-1, VLA-4) on the migrating T cell (Step 4). Finally, after reaching the tumor microenvironment, CD8^+^ T cells form an immunological synapse with tumor cells and kill the malignant cells via the release of cytotoxic granules (Step 5).

DCs orchestrate immune responses and it is now widely appreciated that DCs also play a crucial role in regulating the host immune responses to cancer. Indeed, DCs have been found in various types of tumors both in humans and mice (2, 135–140). In the tumor microenvironment, many tumor cells die naturally or as a result of anti-cancer therapies such as chemotherapy and thus DCs often interact with dying tumor cells enabling them to acquire tumor antigens (141). In addition, immature DCs can also interact with live cells, including other immature or mature DCs and acquire tumor antigens by transferring parts of plasma membrane and intracellular proteins in a process termed “nibbling” (142). Further, immature DCs can also directly interact with live tumor cells and acquire tumor antigens by nibbling (143). Following antigen capture, DCs will ultimately become activated and migrate to the draining lymph node via the lymphatic vessels to present tumor-antigens to T and B cells (141) (Figure 3).

Adhesion receptors on DCs are involved in many of the processes involved in DC-mediated anti-tumor responses. Several receptors such as αVβ5-integrin expressed on immature DCs are involved in the interaction with and phagocytosis of dying cells (144, 145). In addition, given that dying cells often become opsonized by complement component iC3b, DCs can also interact with dying tumor cells via the β2-integrins Mac-1 and αXβ2 (146, 147). However, as described above, β2-integrins often have anti-inflammatory effects in myeloid cells such as DCs, and these interactions lead to suppression of DC activation and, thus, tolerance. Further, since inflammation of various levels has often been associated with tumor development (148), and ICAM-1 expression is up-regulated in lymphatic vessels during inflammation (94), interaction between Mac-1 and ICAM-1 expressed on DCs and inflamed lymphatic endothelium, respectively, may lead to decreased ability of DCs to activate T cells (87). Therefore, integrins on DCs may be involved in the uptake of dying tumor cells via adhesion receptors such as αVβ5-integrin, and in the subsequent initiation of DC-mediated anti-tumor responses. However, β2-integrins expressed on DCs may instead be involved in suppressing DC function. How these processes influence the anti-tumor responses mediated by DCs *in vivo* is currently unclear.

Following tumor-antigen capture, DCs need to enter the lymphatic vessels in order to migrate to the draining lymph node to prime tumor-specific T cells. The role of integrins and other adhesion molecules in the migration of DCs to lymphatic vessels is currently under debate. Ma et al. elaborated anti-ICAM-1 or anti-LFA-1 mAbs effectively inhibiting antigen-bearing epidermal DCs from migrating to regional lymph nodes *in vivo* (149). Studies conducted by Xu et al. further demonstrated the importance of ICAM-1 expression on lymphatic endothelium for the migration of hapten-bearing Langerhans cells into the draining lymph node (150). In addition, Johnson et al. demonstrated the up-regulation of ICAM-1 and VCAM-1 on dermal lymphatic endothelial cells at the presence of inflammatory cytokines which then mediated DC trafficking into the lymph nodes via afferent lymphatic vessels (151). However, according to Grabbe et al., hapten-bearing DC migration from the blood to inflamed skin or from skin into the regional lymph node is similar in β2 (CD18) deficient mice compared to WT mice suggesting that β2-integrins are not required for DC migration into the regional lymph nodes (152). Furthermore, Morrison et al. reported that a mutation in β2 which disrupts the integrin/kindlin interaction and thereby renders the integrin inactive, resulted in increased rather than decreased DC migration to peripheral lymphoid organs through an effect on gene transcription in these cells (37). Accordingly, Podgrabinska et al. further showed that the rate of antigen-bearing DC migration into the lymph nodes was similar between ICAM-1 deficient mice and WT mice (87). Interestingly, according to Thompson et al. naïve tumor-specific CD8^+^ T cells can also become activated and gain effector cell phenotype directly at the tumor site, suggesting that cross-presenting DCs are also able to prime CD8^+^ T cells in the tumor. These results indicate that DC migration into lymph nodes may not even be completely necessary for DC-mediated anti-tumor responses (153). In conclusion, as the role of β2-integrins in DC migration into lymph nodes is currently unclear, whether β2-integrins on DCs influence DC migration and anti-tumor responses in humans is currently not known and requires further study.

In contrast to the unclear role of β2-integrins in DC biology and DC-mediated anti-tumor responses, the αE-integrins may promote DC anti-tumor responses. Several studies have shown that mouse DCs expressing the integrin αE (CD103) are superior in promoting anti-tumor T cell responses (154–157). Indeed, αE^+^ DCs are the main cell population carrying tumor-antigens into the draining lymph node and critical for effective anti-tumor CD8^+^ T cell priming both *in vitro* and *in vivo*. Further, increased tumor growth is observed in the absence of αE^+^ DCs in mouse models of cancer (154). In humans, high αE^+^/αE^−^ ratio has been significantly associated with increased overall survival in various malignancies including breast cancer, head-neck squamous cell carcinoma and lung adenocarcinoma (154). However, the expression of CD141 (BDCA-3, thrombomodulin), rather than αE, has been thought to mark human DC population functionally equivalent to mouse αE^+^ DCs (158). Indeed, Jongbloed et al. and Bachem et al. demonstrated that CD141^+^ DCs were superior in their capacity to cross-present various antigens to CD8^+^ T cells compared to other DC populations (159, 160). Accordingly, a study assessing gene-signatures also associated high intra-tumoral levels of CD141^+^ DCs with better overall survival in melanoma patients (155). Increased disease-free survival among patients with aggressive triple-negative breast cancer was also associated with gene signature specific for CD141^+^ DCs (161).

In conclusion, although integrins play a key role in DC biology, the role of cell adhesion molecules in DC-mediated anti-tumor responses is still unclear and clearly requires further study, especially in human patients.

### Integrins and Other Adhesion Molecules Affect the Development and Tumor-Infiltration of Regulatory Cells

Tregs, defined as CD4^+^CD25^hi^CD127^lo^ or CD4^+^CD25^+^Foxp3^+^ in humans and mice, respectively, are critical in maintaining peripheral tolerance (162). Tregs are able to suppress the effector function and proliferation of various cell populations including T cells, B cells, NK cells, DCs and macrophages by secreting inhibitory molecules such as TGFβ and IL-10, by direct cell-cell contact or by indirect mechanism via antigen presenting cells (APCs) (162–167). For their suppressive function, it is crucial that Tregs are able to migrate to the site of inflammation and thus Tregs express high levels of cell adhesion molecules including ICAM-1, L-selectin, P-selectin, and VLA-4 (168). Interestingly, some adhesion molecules, particularly β2-integrins, also play an important role in Treg development and function (77, 169–171). Wang et al. showed that reduced CD18 (β2) expression in mouse Tregs disrupted the interactions between Tregs and DCs which led to poor Treg proliferation and decreased ability to produce TGFβ1 and thus decreased suppressive function *in vitro* compared to WT Tregs (169). In concordance with *CD18*^−/−^ mice, also *LFA-1*^−/−^ mice showed reduced Treg numbers in secondary lymphoid organs and decreased conversion rate of conventional CD4^+^ T cells into Tregs in the periphery (170). In addition, LFA-1 deficient Tregs failed to suppress CD4^+^ T effector cells *in vitro* and were unable to prevent disease development in experimental colitis model *in vivo*. However, on the contrary to *CD18*^−/−^ mice, *LFA-1*^−/−^ mice showed increase in Treg numbers in the thymus suggesting that in addition to LFA-1, other β2-integrin(s) also affects Treg development. Besides β2-integrins, also L-selectin and integrin αE (CD103) have been associated with Treg function. High expression of L-selectin has been shown to mark Tregs with more potent ability to inhibit graft-versus-host disease (GVHD) and bone marrow (BM) graft rejection in mice compared to L-selectin^lo^ Tregs (172) and αE^+^ Tregs have been shown to be more effective in suppressing acute inflammatory reactions in the induced SCID colitis model and antigen-induced arthritis model compared to αE negative Tregs (173, 174).

Given that Tregs suppress the effector function of various immune cells, they can also inhibit immune responses against cancer cells and thus promote tumor growth (6). Further, the presence of Tregs in the tumor microenvironment can present a major obstacle for successful immunotherapy. Indeed, high number of tumor-infiltrating Tregs has been associated with poor prognosis in several malignancies (4, 175). Conditional depletion of Tregs has been shown to increase anti-tumor immunity in mouse models of cancer suggesting that targeting Tregs could be beneficial approach for cancer patients (176–178). However, the lack of Treg-specific cell surface markers presents a major challenge for this task. Further, targeting Tregs specifically in the tumor microenvironment in order to prevent harmful systemic immune reactions present even a greater challenge. Interestingly, Anz et al. discovered that integrin αE (CD103) is expressed at significantly higher levels in tumor-infiltrating Tregs in several mouse cancer models compared to other peripheral Tregs (90% in CT26-infiltrating Tregs vs. 20% in splenic Tregs) due to increased levels of TGFβ in the tumor microenvironment (179). In addition, αE^+^ Tregs displayed significantly more suppressive phenotype *in vitro* compared to αE negative Tregs. However, given the high expression also on anti-tumorigenic DCs and CD8^+^ T cells, αE seems to be an unsuitable target for cancer immunotherapy.

MDSCs and tumor-associated macrophages (TAMs) also represent cell populations capable of efficient suppression of natural anti-tumor immunity. MDSCs are a heterogeneous population of cells consisting of immature myeloid cells and myeloid progenitor cells (180). As other leukocytes, they are generated in the BM but during pathological conditions such infection or cancer they fail to differentiate into mature DCs, macrophages or granulocytes which leads to accumulation of immature myeloid cells with highly immunosuppressive phenotype (180, 181). MDSCs can be divided into different sub-populations in humans (CD11b^+^CD14^−^CD15^+^, CD11b^+^CD14^−^CD66b^+^ and CD11b^+^CD14^+^HLA-DR^−/lo^CD15) and mice (CD11b^+^Ly6G^+^Ly6C^lo^ and CD11b^+^Ly6G^−^Ly6C^hi^) based on their phenotypic differences but they all share myeloid origin and the ability to strongly suppress T and NK cell activity (180–182). MDSCs are recruited into the tumor site by the malignant cells by increasing the level of various soluble factors including IL-6, GM-CSF, TGFβ, VEGF and chemokines such as CCL2 and CCL5, in the tumor microenvironment (180, 183). After reaching the tumor site, MDSCs efficiently suppress the anti-tumor immunity by various mechanisms such as by depleting T cell nutrients such as L-arginine, by producing reactive oxygen species (ROS) and nitric oxide (NO) and by promoting the development of Tregs. Indeed, several studies have reported an increase in anti-tumor immunity following depletion or suppression of MDSCs in mouse models of cancer (184–186). Among human patients, an increase in MDSCs has been observed in the tumor tissue and peripheral blood in many cancer types (187–191). Further, an increase in MDSCs in the peripheral blood has been associated with poor prognosis in patients with various solid tumors (5, 192). Interestingly, Jin et al. reported VLA-4 being responsible for recruitment of circulating monocytes and macrophages into the tumor site (193). Later it was found that tumor growth was significantly suppressed in mice lacking activated form of VLA-4 and in WT mice treated with mAbs targeting VLA-4 (194). In addition, tumors derived from these mice had significantly reduced frequencies of total CD11b^+^Gr1^+^ myeloid cells but increased numbers of CD8^+^ T cells and mature (CD80^+^) DCs. *In vitro* IL-4 stimulated macrophages derived from mice lacking activated form of VLA-4 also showed decreased levels of *Il10, Tgfb1*, and *Arg1* mRNAs but increased levels of mRNAs encoding for IFNγ and IL-12 compared to WT macrophages. These results suggested that VLA-4 regulates MDSC trafficking into the tumor site and also promotes myeloid cell polarization toward immune suppressive phenotype, inhibits anti-tumor immunity and thus promotes tumor growth.

Mac-1 is highly expressed in myeloid cells and plays a key role in various myeloid cell functions including migration, phagocytosis, and chemotaxis (195). In addition, given that mAbs targeting Mac-1 have been shown to decrease myeloid cell tissue infiltration and inflammation (196), targeting Mac-1 could also reduce the recruitment of suppressive myeloid cells into the tumor site. Indeed, Zhang et al. demonstrated that CD11b (αM-chain of Mac-1) deficiency reduced intestinal tumor growth in mice by reducing myeloid cell trafficking to the tumor microenvironment (197). Further, CD11b blockade also decreased myeloid cell recruitment into tumors in immune deficient mice bearing human squamous cell carcinoma xenografts and thus improved the anti-tumor responses to radiotherapy (198). However, as Mac-1 has been shown to play other roles in immunity than simply affecting cell recruitment, e.g., in immune suppression, it is possible that CD11b blockade also has other, as yet unrecognized effects on anti-tumor immunity.

### Integrins and Other Cell Adhesion Molecules Also Have Cell Intrinsic Effects on Tumor Cells

Integrin expression on tumor cells themselves has been associated with tumor progression and metastasis by increasing the proliferation, survival, migration and invasion of the malignant cells (199). Integrin ligation can promote tumor cell survival by several mechanisms such as by inhibiting p53 and caspase-9 via αV (200) and by increasing anti-apoptotic proteins Bcl-2 and Bcl-X_L_ via αVβ5 and αVβ3 (201). Particularly integrin αVβ3 has been associated with tumor progression in various human malignancies (202–204) and it co-operates with *SRC* oncogene to enhance anchorage-independent tumor growth and promotes lymph node metastases (205, 206). Other integrins have also been shown to co-operate with oncogenes including β4 which amplifies the signaling of ErbB2 to promote mammary tumorigenesis (207). In addition, integrins such as β3 may also function as markers for highly tumorigenic cancer stem cells (208).

Besides on tumor-associated vasculature, aberrant VCAM-1 expression has also been described on many types of tumor cells such as breast, renal and gastric carcinoma cells (97, 209). Up-regulation of VCAM-1 on malignant cells is associated with increased ability to metastasize and recruit tumor-associated monocytes and macrophages. Also ICAM-1 expression has been associated with the malignant potential of tumor cells and has thus been associated with metastases and poor prognosis in several cancers including melanoma, breast, lung and oral cancer (210–213). Given that MUC-1 which is often expressed on tumor cells (214) can also interact with ICAM-1, circulating cancer cells can adhere to endothelial cells which may represent the first step in metastases formation (215). MUC-1-ICAM-1 interaction can then induce cytokine secretion and ICAM-1 up-regulation in the malignant cells thus recruiting macrophages. Indeed, macrophage infiltration has been demonstrated to correlate with ICAM-1 expressing tumor cells in oral squamous cell carcinoma (213). Macrophage-derived cytokines can then further attract neutrophils which secrete proteases promoting extraluminal migration of the malignant cells (215). Further strengthening the role of ICAM-1 in tumor metastasis, tumor cell lines transfected with ICAM-1 showed enhanced invasive capacity and proliferation *in vitro* (211, 213) and ICAM-1 blockade have been shown to decrease tumor cell invasion *in vitro* (211, 216). In addition, an increase in serum levels of soluble ICAM-1 (sICAM-1) has been associated with disease progression, tumor aggressiveness and decreased survival in several malignancies such as melanoma, chronic B-lymphocytic leukemia, lymphoma, and CRC (217–220).

Given the specific expression on hematopoietic cells, LFA-1 plays a role in the development of hematological malignancies such as leukemias and lymphomas. Chronic lymphocytic leukemia (CLL) is the most common leukemia in the Western countries (221). It is characterized by clonal mature B cell accumulation in the BM, blood and other lymphoid tissues and advanced disease often manifests itself as lymphadenopathy, hepatomegaly, splenomegaly, BM failure, and recurrent infections. The malignant CLL cells are highly dependent on lymphoid microenvironment where they interact with and receive survival and proliferation signals from the accessory cells (222). In addition to chemokines, malignant cells require the expression of leukocyte adhesion molecules, particularly LFA-1 and VLA-4, in order to migrate into the lymphoid tissues. Interestingly, Montresor et al. reported differences in integrin signaling in human CLL cells compared to healthy B cells (223). Signaling molecules such as PIP5KC, RAC1, and CDC42 regulating integrin conformational change into the active form in normal lymphocytes showed either none or severely decreased regulatory role in CLL cells. By using live cell imaging, Till et al. further showed that in contrast to LFA-1 expressed on normal human B cells, LFA-1 expressed on CLL cells can be in its active conformation without chemokine induced clustering (224). In addition to aberrant integrin signaling, the expression levels of adhesion molecules on CLL cells usually differ from healthy B cells. Generally, the expression of various adhesion molecules including β2- and β1-integrins, CD54, CD62L, and CD44 is very low on peripheral blood and BM CLL cells compared to cells derived from healthy donors (225). Interestingly, Hartmann et al. demonstrated that CLL cells require the same integrins as healthy B cells in trafficking through the BM, spleen, and lymph nodes (226). On the contrary to normal human B cells, CLL cells were unable to arrest in ICAM-1 expressing endothelium *in vitro* and to migrate to lymph nodes of NOD/SCID mice *in vivo* due to low expression of LFA-1. In addition to low LFA-1 expression, VLA-4 expression was variable on CLL cells but yet significantly reduced compared to healthy B cells. Therefore, the ability of human CLL cells to migrate to the BM of NOD/SCID mice *in vivo* was significantly reduced compared to normal B cells. These results suggested that CLL cell migration to the BM and lymph nodes is decreased due to low expression of LFA-1 and VLA-4 thus causing an accumulation in the blood and spleen. Interestingly, the same study also reported that significantly higher expression of LFA-1 and VLA-4 was detected on CLL cells derived from high-risk patients with unfavorable cytogenetic abnormalities such as trisomy 12, deletion 17p or deletion 11q (226). Increased expression of integrins including LFA-1 and VLA-4 on CLL cells among patients with trisomy 12 has also been reported by others and the increase has been associated with up-regulation of molecules regulating integrin inside-out signaling such as RAP1B and RAPL and with enhanced ligand (ICAM-1, VCAM-1) binding and migration *in vitro* (227, 228). These results suggest that up-regulation of cell adhesion molecules, particularly LFA-1 and VLA-4, could increase CLL cell migration to lymphoid tissues where they would receive more proliferation and survival signals thus leading to more aggressive disease (226, 227). Indeed, higher expression of CD11a (LFA-1 α-chain) on CLL cells has been associated with increase in tumor burden (225) and higher expression of CD49d (VLA-4 α-chain) with disease progression and decreased overall survival (229–231).

Multiple myeloma (MM) is a cancer of the BM caused by malignant, terminally differentiated plasma cells (PCs) (232, 233). It is the second most common hematological malignancy in the world and its symptoms include increased calcium levels, renal insufficiency, anemia, and/or bone lesions. In most cases the malignant plasma cells also secrete monoclonal immunoglobulin proteins (M-proteins) that can be detected in blood and/or urine. Despite the recent improvements in MM therapies, MM is still largely considered to be an incurable disease. As in CLL (222), adhesion molecules play an important role in the interactions between MM cells and accessory cells in the BM thus promoting the proliferation and survival of the malignant cells (234). Indeed, high proportion of human myeloma cells have been shown to express various adhesion molecules including LFA-1, VLA-4, CD44, and ICAM-1 (235–237). LFA-1 expression on myeloma cells in particular has been associated with aggressive disease (236, 237) and to correlate with disease activity (235). Interestingly, by using a mouse MM cell line (5T33MMvt), Asosingh et al. demonstrated LFA-1 playing a role in homotypic cell-cell adhesion and cell proliferation of myeloma cells *in vitro* (238). Further, in contrast to LFA-1 negative cells, only LFA-1 expressing 5T33MMvt cells were able to cause disease *in vivo*. Finally, various adhesion molecules including LFA-1 and VLA-4 have also been associated with drug resistance in MM patients (239).

### Anti-Tumor Therapy Targeting Cell Adhesion Molecules

Given that integrin function has been associated with tumor progression, there has been great interest in targeting integrins in the treatment of cancer. However, since integrins also play diverse roles in immunity and anti-tumor responses, blocking or enhancing the function of these molecules *in vivo* may be difficult.

The majority of drugs targeting integrins in the aim to treat cancer inhibit the function of αV- or β1-integrins (240, 241). Both integrin classes have been shown to be highly expressed in many human malignancies and antagonists targeting αV or β1 indeed suppressed tumor growth in preclinical mouse models (199, 202, 242–248). As a result, there have been multiple clinical trials targeting integrins either as a single therapy or in combination with conventional therapies in treating various human cancers (249–289) (Table 1). However, in contrast to the pre-clinical studies, the results from clinical trials were rather disappointing with αV or β1 antagonists generally showing either no or only week anti-tumor efficacy (Table 1). In one phase II clinical trial with advanced prostate cancer patients, intetumumab (CNTO 95), a mAb targeting multiple αV integrins, in fact resulted in decreased progression-free and overall survival compared to placebo (276). However, it could be possible to improve therapeutic responses of integrin targeting by combining it with other immunotherapies. Indeed, mouse studies conducted by Kwan et al. demonstrated that by combining a fusion protein, consisting of a mouse Fc domain and RGD-binding integrin targeting peptide, with albumin/IL-2 or anti-PD-1 immunotherapy it was possible to enhance anti-tumor immunity and tumor suppression (290).

**Table 1:** Clinical trials targeting integrins.

**Target**	**Compound name**	**Molecule**	**Study phase**	**Malignancy**	**Combination therapies**	**Clinical outcome**	**References**
αV	Intetumumab (CTNO 95)	mAb (fully human IgG1)	Phase 1	Solid tumors	-	One partial response (4,2% of patients)	(256)
			Phase 2	Melanoma	Dacarbazine ± Intetumumab	No significant differences in the efficacy between treatment groups	(271)
			Phase 2	Prostate cancer	Docetaxel + Prednisone ± Intetumumab	All efficacy end points favoured placebo over intetumumab	(276)
			Phase 1	Solid tumors	Bevacizumab + Intetumumab	No tumor responses	(283)
αV	IMGN-388	Antibody-drug conjugate (intetumumab bound to maytansinoid cytotoxic agent DM4)	Phase 1	Solid tumors	-	No evidence of activity	(267)
αV	Abituzumab (EMD 525797; DI17E6)	mAb (humanized IgG2)	Phase 1	Prostate cancer	-	One partial response (3,8% of patients)	(279)
			Phase 1/2	Colorectal cancer	SoC (Cetuximab + Irinotecan) ± Abituzumab	Primary end point was not met, no significant differences in efficacy was observed between treatment groups	(280)
			Phase 2	Prostate cancer	Luteinizing hormone-releasing hormone agonist/antagonist therapy ± Abituzumab	No significant differences between treatment groups	(286)
αV/α5β1	GLPG-0187	Small molecule inhibitor	Phase 1	Glioma and other solid tumors	-	No tumor responses	(285)
αVβ3/α5β1	ATN-161	Small molecule inhibitor	Phase 1	Solid tumors	-	No objective responses	(251)
αVβ3/αVβ5	Cilengitide (EMD121974)	Small molecule inhibitor	Phase 2	Pancreatic cancer	Gemcitabine ± Cilengitide	No differences in clinical efficacy between the treatment groups	(254)
			Phase 1	Malignant glioma	-	Shows clinical activity	(257)
			Phase 1	Refractory brian tumors	-	Preliminary evidence of clinical activity	(260)
			Phase 2	Glioblastoma	-	Modest antitumor activity	(261)
			Phase 1/2a	Glioblastoma	Temozolomide + radiotherapy + Cilengitide	Promising activity compared to historical controls	(266)
			Phase 2	Prostate cancer	Luteinizing hormone-releasing hormone therapy + Cilengitide	Modest clinical effect	(269)
			Phase 2	Glioblastoma	Surgery + Cilengitide	Modest antitumor activity	(272)
			Phase 2	Melanoma	-	Minimal clinical efficacy	(273)
			Phase 1	Solid tumors	-	No objective responses	(274)
			Phase 3	Glioblastoma	Temozolomide + radiotherapy ± Cilengitide	Addition of cilengitide did not improve outcomes	(277)
			Phase 2	Squamous cell carcinoma of the head and neck	Cisplatin + 5-Fluorouracil + Cetuximab ± Cilengitide	No significant differences between the treatment groups	(278)
			Phase 1	Glioblastoma	Cediranib + Cilengitide	Response rates do not warrant further development of this combination	(281)
			Phase 2	Glioblastoma	Temozolomide + Radiotherapy ± Cilengitide	No firm conclusions regarding clinical efficacy were able to be made	(282)
			Phase 2	Non-small-cell lung cancer	Cetuximab + platinum-based chemotherapy (Cisplatin/Vinorelbine or Cisplatin/Gemcitabine) ± Cilengitide	Potential clinical activity	(284)
			Phase 2	Glioblastoma	Cilengitide + radiotherapy + Temozolomide + Procarbazine	Response rates do not warrant further development of this combination	(287)
			Phase 1	Solid tumors	Paclitaxel + Cilengitide	Antitumor activity was observed	(289)
αVβ3	Etaracizumab (abegrin; MEDI-522)	mAb (humanized IgG1)	Phase 1	Solid tumors	-	No objective responses	(250, 259)
			Phase 2	Melanoma	Taracizumab ± Dacarbazine	Etaracizumab had no tumor response when given as a single treatment; Etaracizumab + Dacarbazine similar to historical data for Dacarbazine alone; phase 3 study not reasonable	(264)
αVβ3	Vitaxin (MEDI-523)	mAb (humanized IgG1)	Phase 1	Solid tumors	-	Potential activity	(249)
α5β1	Volociximab (M200)	mAb (humanized chimeric IgG4)	Phase 2	Renal cell cancer	-	Best outcome was stable disease	(253)
			Phase 2	Melanoma	Dacarbazine + Volociximab	Best outcome was stable disease	(252)
			Phase 2	Pancreatic cancer	Gemcitabine + Volociximab	One partial response (5% of patients)	(255)
			Phase 2	Melanoma	-	Insufficient clinical activity to proceed to the second stage of the trial	(258)
			Phase 1	Solid tumors	-	Preliminary activity was observed in two patients (9,5% of patients)	(262)
			Phase 2	Ovarian and peritoneal cancer	Doxorubicin ± Volociximab	No differences between the treatment groups	(263)
			Phase 2	Epithelial ovarian and peritoneal cancer	-	Insufficient clinical activity	(268)
			Phase 1b	Non-small-cell lung cancer	Carboplatin + Paclitaxel + Volociximab	Best outcome was partial response (24% of patients)	(275)
α2	E-7820	Small molecule inhibitor	Phase 2	Colorectal cancer	Cetuximab + E-7820	Objective response rate 3.6%	(265)
			Phase 1	Advanced solid tumors	-	Best outcome was stable disease	(270, 288)

Due to the specific expression on hematopoietic cells, β2-integrins have mainly been aimed to target to treat inflammatory diseases including liver fibrosis (291) and autoimmune diseases, including arthritis (292), and psoriasis (293). In pre-clinical studies, LFA-1 small-molecule antagonist (BMS-587101) inhibited LFA-1-mediated T cell adhesion to endothelial cells, Th1 cytokine production, and T cell proliferation *in vitro* and also inhibited inflammation *in vivo* (292). In the context of cancer, β2-integrins, mainly LFA-1, would be an attractive target to treat hematological cancers such as leukemias and lymphomas (294, 295). In addition, LFA-1 small molecule antagonists have also demonstrated anti-tumor efficacy against solid tumors in mice (296). However, given that LFA-1 promotes T cell activation and migration, blocking the function of LFA-1 may in fact increase the risk of malignancies and infections (297). Indeed, anti-LFA-1 therapy has been associated with rare but severe systemic adverse events such as immune-mediated thrombocytopenia and hemolytic anemia (298). Further, in 2009, after more than 45,000 psoriasis patients had been treated with efalizumab (humanized anti-CD11a mAb), the drug was withdrawn from the market due to three confirmed cases of fatal viral-based multifocal leukoencephalopathy (PML) (297, 299). One possibility to harness the anti-tumor efficacy of LFA-1 blockade without inducing severe adverse events would be to target LFA-1 specifically on tumor cells. Indeed, Cohen et al. demonstrated that by using bispecific antibody which simultaneously targets LFA-1 and a tumor specific antigen it was possible to specifically block LFA-1-mediated tumor cell adhesion without affecting immune responses in mice (294). In addition, it is also noteworthy that since activation of LFA-1 has been shown to regulate the activity of VLA-4, drugs targeting LFA-1 may also affect the function of other integrins (300).

Given the lack of anti-tumor efficacy and in the case of LFA-1 blockers, the severity of adverse events, it could be more feasible to target the integrin ligands on tumor vessels than the integrins themselves in order to enhance T cell infiltration to the tumor site. As discussed before, previously E-selectin negative tumor-associated vessels in human SCC samples up-regulated E-selectin following treatment with imiquimod (TLR-7 agonist) which resulted in CD8^+^ T cell influx into the tumor and tumor regression (129). Also systemic thermal therapy (STT) induced the activation of IL-6 trans-signaling causing up-regulation of ICAM-1 on tumor vascular endothelium and thus increased the homing of adoptively transferred CD8^+^ T cells into tumors in mouse models of cancer (125). Further, CpG-ODN (oligodeoxynucleotides (ODN) with cytocine-guanine-rich (CpG) motifs) vaccination caused up-regulation of ICAM-1 and VCAM-1 on tumor-associated blood vessel endothelia leading to strong tumor-infiltration of adoptively transferred tumor-specific T cells and tumor suppression in mouse model of pancreatic islet cell carcinoma (301). These results demonstrated that by increasing inflammatory signals in the tumor microenvironment it could be possible to enhance T cell infiltration into tumors and thus T-cell mediated tumor cell killing. The most straight forward way to increase inflammatory signals would be to administer inflammatory cytokines such as TNFα or IFNγ systemically (302). However, this could lead to severe adverse events as has been demonstrated with the systemic administration of TNFα which was associated with risk of septic shock syndrome leading to multi-organ failure (303). Delivery of inflammatory cytokines or other inflammatory stimuli directly to the tumor site could reduce the risk of side effects (302). Further, systemic administration of TNFα coupled to ACDCRGDCFCG-peptide, a ligand for αVβ3-integrin, has enabled targeting the tumor vasculature and inducing anti-tumor effects in tumor-bearing mice (304). Given that angiogenic factors have been shown to cause decrease in ICAM-1 and VCAM-1 expression on tumor associated vessels (105, 110), targeting angiogenesis could also increase T cell infiltration into tumors. Indeed, treatment with angiogenesis inhibitor anginex significantly up-regulated the expression of VCAM-1 and E-selectin on tumor blood vessel endothelial cells resulting in increase in tumor-infiltrating leukocytes and suppression of tumor growth in mouse models of cancer (305). Further, VEGF blockade significantly increased tumor infiltration of adoptively transferred T cells thus promoting tumor suppression in mice (306).

Among therapies involving adoptively transferred cells, chimeric antigen receptor (CAR) T cell therapy has shown promise in cancer treatment due to measurable responses in several clinical trials across various malignancies (307–310). Given that CAR T cells are usually constructed by joining an antigen-recognition moiety such as single chain antibody (scFv) to TCR/CD3 complex and by adding costimulatory domain such as CD28 or CD137 to improve cell survival and proliferation, CAR T cell therapy has several advantages to conventional T cell therapy such as higher antigen binding affinity and independency of MHC expression on tumor cells (311). However, the major obstacle for CAR T cell therapy is often the inability of the transferred cells to migrate and extravasate into the tumor (312). One way to overcome this obstacle could be to generate a CAR T cell with specificity to tumor-associated vasculature instead of the tumor cells themselves (313). Fu et al. constructed a CAR T cell containing a peptide sequence for echistatin (T-eCAR) which has a high affinity to the integrin highly expressed on tumor vasculature endothelium, αVβ3 (313). Indeed, T-eCAR cells efficiently lysed αVβ3 expressing human umbilical vein endothelial cells and tumor cells *in vitro*. Further, these cells led to extensive bleeding in the tumor tissue and thus tumor growth suppression while sparing normal tissues in mice. In addition to CAR T cells, integrins expressed on the tumor vasculature could also be utilized in order to target drug-delivering nanoparticles to the tumor tissue more efficiently (199). Murphy et al. demonstrated that doxorubicin containing, αVβ3-targeted lipid nanoparticles effectively targeted tumor vessels leading to their apoptosis in mouse models of cancer (314). Moreover, αVβ3-targeted nanoparticles showed anti-metastatic effects in spontaneous metastases models without causing adverse events associated with systemic administration of free doxorubicin.

It may also be possible to enhance the anti-tumor response of adoptively transferred cells by increasing LFA-1 function in the cells. Simply “locking” LFA-1 in an active state (through mutation) is unlikely to be successful, as T cells expressing such mutated integrins are actually deficient in T cell migration, which requires dynamic modulation of integrin activity (315). Instead, indirect approaches may be required. Indeed, mutation of a phosphorylation site in VLA-4 leads to increased LFA-1 activity (through integrin transregulation) and enhanced tumor immunity (316). Also, modulating LFA-1 activation status through engineering of ALCAM increases tumor rejection of brain tumors (317).

### Conclusions and Future Perspectives

Taken together, adhesion molecules play vital roles in the function of the immune system both in health and disease. During cancer development, adhesion molecules, particularly integrins, mediate crucial functions in nearly every step of the anti-tumor response including in tumor antigen uptake, activation of tumor-specific T cells, leukocyte trafficking into the tumor site and tumor cell killing. However, malignant cells can also utilize cell adhesion molecule pathways to promote tumor growth. Expression of various integrins on tumor cells promotes tumor cell proliferation, survival and metastases while increased secretion of angiogenic molecules causes down-regulation of adhesion molecules on tumor-associated blood vessels and thus prevents immune effector cell infiltration into the tumor. Tumor cells also recruit regulatory cells such as Tregs and MDSCs which express high levels of integrins enabling them to reach the tumor site. The main cell adhesion molecule-mediated events promoting tumor growth are listed in Table 2.

**Table 2:** Adhesion molecule-mediated events promoting tumor growth.

**Event**	**Type of tumor**	**Adhesion molecule-mediated mechanisms operating in the tumor microenvironment**	**Consequence for tumor progression**	**References**
1	Solid	Increased secretion of angiogenic factors by the tumor cells reduces the expression of various adhesion molecules including ICAM-1/2, VCAM-1 and E-selectin in tumor-associated endothelial cells	Leukocytes in blood are unable to extravasate to the tumor site (endothelial anergy)	(102, 103, 105, 110–112)
2	Solid	Dying tumor cells become opsonized with iC3b	DCs interact with dying tumor cells via β2-integrins Mac-1 and CD11c/CD18 leading to suppression of DC activation and tolerance	(146, 147)
3	Solid	High expression of adhesion molecules including ICAM-1, VLA-4 and L-selectin on Tregs	Affects Treg trafficking possibly enabling them to reach the tumor site where they suppress effector T cells leading to tumor evasion of the immune system	(168)
4	Solid	High expression of VLA-4 and CD11b on myeloid cells	Myeloid cells are able to reach the tumor site and promote angiogenesis and tumor growth	(193, 195–197)
5	Solid	Expression of various integrins including αVβ3, ICAM-1 and VCAM-1 on tumor cells	Increase in tumor cell proliferation, survival and invasion, recruitment of Tumor Associated Macrophages (TAMs) which allows evasion of the immune system	(97, 201–206, 209–213)
6	Solid	Expression of MUC-1 on tumor cells, which is able to bind to ICAM-1 in endothelial cells	Tumor cells are able to cross the endothelial barrier, which promotes metastasis	(214, 215)
7	Hematological	Upregulation of LFA-1/VLA-4 expression on tumor cells which are able to bind to ICAM-1/VCAM-1 in endothelial cells	Tumor cells are able to cross the endothelial barrier and migrate to lymphoid tissues to receive more proliferation and survival signals promoting tumor progression	(222, 225–227, 229–231, 235–237)

Immunotherapy including immune checkpoint blockade and CAR T cells has revolutionized the field of cancer therapy during the last decades. Given the various roles in tumor development, integrins also seem like promising targets for cancer therapy. However, clinical trials targeting integrins directly on malignant cells have shown disappointing results with low therapeutic efficacy. Rather, increasing the expression or function of β2-integrins on immune cells or their ligands on tumor-associated blood vessels and enhancing anti-tumor responses may represent a more efficient approach. In addition, considering that still a considerable number of patients do not benefit from current forms of immunotherapy due to the inability of T cells to access the tumor microenvironment, enhancing β2-integrin function could open a possibility to overcome this impediment. For this and also to prevent harmful treatment-related adverse events, it is vital to fully understand the various functions of β2-integrins and how their expression and function are regulated.

## Author Contributions

All authors contributed to writing the manuscript, with HH and SF playing leading roles in manuscript planning and writing. ML and CG wrote part of the text. ML made the figures.

### Conflict of Interest Statement

The authors declare that the research was conducted in the absence of any commercial or financial relationships that could be construed as a potential conflict of interest.
